# Identification of pathognomonic purine synthesis biomarkers by metabolomic profiling of adolescents with obesity and type 2 diabetes

**DOI:** 10.1371/journal.pone.0234970

**Published:** 2020-06-26

**Authors:** Jennifer Concepcion, Katherine Chen, Rintaro Saito, Jon Gangoiti, Eric Mendez, Maria Eleni Nikita, Bruce A. Barshop, Loki Natarajan, Kumar Sharma, Jane J. Kim

**Affiliations:** 1 Department of Pediatrics, University of California San Diego, La Jolla, CA, United States of America; 2 Rady Children’s Hospital, San Diego, CA, United States of America; 3 Keio University, Yamagata, Japan; 4 Department of Family Medicine and Public Health, University of California San Diego, La Jolla, CA, United States of America; 5 Center for Renal Precision Medicine, Department of Medicine, University of Texas Health San Antonio, San Antonio, TX, Unied States of America; University of Illinois, UNITED STATES

## Abstract

The incidence of type 2 diabetes is increasing more rapidly in adolescents than in any other age group. We identified and compared metabolite signatures in obese children with type 2 diabetes (T2D), obese children without diabetes (OB), and healthy, age- and gender-matched normal weight controls (NW) by measuring 273 analytes in fasting plasma and 24-hour urine samples from 90 subjects by targeted LC-MS/MS. Diabetic subjects were within 2 years of diagnosis in an attempt to capture early-stage disease prior to declining renal function. We found 22 urine metabolites that were uniquely associated with T2D when compared to OB and NW groups. The metabolites most significantly elevated in T2D youth included members of the betaine pathway, nucleic acid metabolism, and branched-chain amino acids (BCAAs) and their catabolites. Notably, the metabolite pattern in OB and T2D groups differed between urine and plasma, suggesting that urinary BCAAs and their intermediates behaved as a more specific biomarker for T2D, while plasma BCAAs associated with the obese, insulin resistant state independent of diabetes status. Correlative analysis of metabolites in the T2D signature indicated that betaine metabolites, BCAAs, and aromatic amino acids were associated with hyperglycemia, but BCAA acylglycine derivatives and nucleic acid metabolites were linked to insulin resistance. Of major interest, we found that urine levels of succinylaminoimidazole carboxamide riboside (SAICA-riboside) were increased in diabetic youth, identifying urine SAICA-riboside as a potential biomarker for T2D.

## Introduction

There has been an alarming rise in the prevalence of obesity and type 2 diabetes in children in the past 3 decades. [1] At present, the clinical management of diabetic youth is largely based on therapies used in adults. However, we cannot assume that the basic pathophysiology of type 2 diabetes is similar in pediatric and adult populations. Recent studies show that type 2 diabetes may have a more aggressive course in youth. For example, the destruction of pancreatic β cells occurs at a rate almost four times higher than in adults. [2] Complications such as hypertension, nephropathy [3] and retinopathy [4] appear faster in children than in adults. Moreover, a much higher proportion of adolescents fail to respond to metformin when compared to adults. [5] After more than 20 years, the optimal treatment of type 2 diabetes in childhood remains largely unknown.

Elevated plasma BCAAs have been observed in obese, insulin resistant human adults [6, 7] and adolescents [8–11], postulated to be due to impaired BCAA catabolism. [12, 13] Most prior studies in adolescents have examined obese subjects with insulin resistance, but very few have addressed how metabolic pathways are altered in youth with recent onset type 2 diabetes. Moreover, little is known about urine metabolite patterns in these populations. In contrast to plasma metabolomics that present a relatively narrow subset of compounds related to intermediary metabolism, a wider range of organic and amino acids can be measured in urine [14] as the kidney is responsible for concentrating a variety of metabolites that are secreted into the urine, and the fluctuations in plasma concentration are time-averaged in urine. [15, 16] Therefore, measurement of urine metabolites may provide greater insight into the metabolic variations that underlie these disease states. Previous work from our group indicated a metabolomic signature in patients with diabetic kidney disease who all had reduced eGFR. [17] We are not aware of any prior studies which examined urine metabolomic profiles in adolescents with obesity or type 2 diabetes.

In this study, we employed a targeted, quantitative mass spectrometry-based approach to generate unique urine and plasma metabolite signatures that differentiate obese youth with and without type 2 diabetes, as a first step toward identifying novel biomarkers that predict the development of diabetes in children. All diabetic subjects were within 2 years of their initial diagnosis in an attempt to capture early-stage disease prior to declining renal function. We hypothesized that subjects with type 2 diabetes would be distinguished by a discrete set of urine metabolites including altered branched chain amino acid metabolites. We used liquid chromatography coupled to tandem mass spectrometry (LC-MS/MS) and bioinformatic analysis to investigate differences in metabolites concentrations among NW, OB and T2D adolescents, examine differences in excretion, and correlate metabolites to clinical markers of diabetes and renal function.

## Research design and methods

### Study design and participants

The study sample consisted of 3 groups of children (n = 30 children per group) ages 13–19 years old recruited from Rady Children’s Hospital in San Diego, CA (ClinicalTrials.gov/NCT02145572). The groups were: obese children with early type 2 diabetes (T2D), obese children without diabetes (OB), and healthy, age- and gender-matched normal weight controls (NW).

Obesity was defined as a BMI >95^th^ percentile for age/gender according to CDC norms, and diabetes was defined by current American Diabetes Association criteria. For eligibility, patients with type 2 diabetes needed to be within 2 years from their diagnosis and show absence of pancreatic autoimmunity. Subjects were excluded from participation if there was evidence of renal failure (estimated GFR <60mL/min), use of medications that would affect glycemia (such as steroids), and other significant organ system illness or condition. The study was approved by the University of California San Diego Institutional Review Board.

This cross-sectional study required each subject to provide a 24-hour urine collection and same day fasting blood sample. A 24-hour rather than spot urine collection was obtained in order to more accurately assess compounds with very short half-times; these compounds would not be accurately represented in a single plasma or spot urine sample due to their rapid absorption and elimination. [18] Routine clinical biochemistry was performed at the Rady Children’s Hospital laboratory and included fasting glucose, insulin, C-peptide, HbA1C, urine microalbumin, AST, ALT, and a fasting lipid panel. Creatinine clearance was calculated from an estimated eGFR (eGFR) using the “Bedside Schwartz” equation. Urine and plasma samples for metabolomic analysis were stored in our biorepository at -80°C until all subjects’ samples were collected and ready for MS measurement.

### Metabolomics

Samples were assayed using an adaptation of Gerstman et al.. [19] For normalization purposes, the volumes of urine equivalent to 0.2 micromole of creatinine, pre-measured using the Jaffe colorimetric method, or 100μL of plasma, were extracted in 80% ice-cold methanol containing stable-isotope labeled internal standards (stable-isotope dilution (SID)), after vortex-mixing, incubation for 30 minutes at -20°C and centrifugation at 17,136 x*g* at 5°C. Supernatants were evaporated to dryness in a centrifugal evaporator at 36°C (Savant SPD121P Speed Vac concentrator, Thermo Fisher, Asheville, NC, USA) and reconstituted in 100μL 10% methanol in water + 0.1% formic acid by means of consecutive vortexing, orbital shaking and sonication.

Samples were then analyzed by liquid chromatography-electrospray tandem mass spectrometry (LC-ESI-MS/MS) (Sciex API4000, Foster City, CA, USA) using both positive- (acylcarnitines, amino acids, purines and pyrimidines, biogenic amines) and negative-(acylglycines, organic acids and sugar and sugar alcohols formyl adducts) mode-specific parent to daughter transitions, individually tailored by infusing the authentic standards and monitored by scheduling them at the corresponding retention times. Collision gas was set to 12 psi, curtain gas to 30 psi; nebulizer and heater gases were 40 and 60 psi, respectively. Source temperature was 500°C. Ion voltages were -4,500 and 5,500 volts, in negative and positive ion modes, respectively.

After order randomization, 5μL were injected in the analytical system (CTC Analytics HTC PAL autosampler, Raleigh, NC, USA). Chromatographic separations were conducted on a 3 μm ACE C18-PFP reversed-phase HPLC column (Mac-Mod analytical, Chadds Ford, PA, USA) using an Agilent 1200 Series pump (Agilent Technologies, Waldbronn, Germany) equipped with an in-line degasser at 0.3 mL/min flow-rate and at 25°C, by means of a simple binary acetonitrile (B) partitioning in water gradient, both containing 0.1% formic acid. The gradient for negative ionization runs was 0%B the first 18.6 minutes, then ramped up to 17%B by 29.2 minutes, and 100%B at 34 minutes, where it was rinsing for an additional 3 minutes. Total run time was 56 minutes. The gradient for positive ionization runs was 3%B for the first 3 minutes, then 40%B by 16 minutes, and 100%B at 26 minutes where it stayed for two additional minutes. Total run time was 45 minutes.

Metabolite concentrations were calculated using the authentic standard dissolved in synthetic urine [20] supplemented with 50mg/L recombinant human albumin (rHSA) (Sigma-Aldrich. St. Louis, MO, USA), instead of peptone L37 and yeast extract, without endogenous creatinine, lactic, citric and uric acids, but with those compounds added as individual calibrators. We prepared curves in six to eight non-zero levels calibrator levels, spanning the physiological range, and using statistical weighting proportional to the reciprocal of the concentration, to compensate for different variances at low concentration, confirming correlation coefficients of 0.99, or higher. Quantification was conducted using MultiQuant 2.1 software (Sciex, Foster City, CA, USA). All standards and their suppliers are listed in S1 Table. Information on all precursor-product ion transitions in negative and positive ionization modes are listed in S2 Table.

### Statistical analysis

To examine subject characteristics, we used ANOVA with Bonferroni post hoc correction for quantitative variables and χ^2^ test for categorical variables. Data are presented as mean ± SEM unless otherwise indicated. Statistical significance was set at *P* < 0.05. The analyses were performed using Prism (version 6.0h, GraphPad Software, La Jolla, CA).

The assessment of differences in individual metabolite levels among three sample groups (NW, OB and T2D) was performed using ANOVA and Principal Components Analysis (PCA). The levels of detected metabolites were log2-transformed before doing ANOVA or PCA. If a metabolite for a subject was not detected, then half the value of the smallest detected metabolite level among all measured samples for that metabolite was imputed so that the metabolite level could be log-transformed. If the metabolite was not detected from more than one-third of the subjects, then we used a binary categorization for this metabolite as detected versus not, followed by Fisher’s exact test to test for significant differences in proportions with detectable levels of the metabolite among the three groups.

The calculated ANOVA (or Fisher exact) p-values were then subjected to multiple testing correction using the Benjamini-Hochberg (BH) method (*P* < 0.05 was set as the threshold). Differences between each pair of sample groups were further tested with Tukey’s range test (*P* < 0.05). Similarly, ANOVA was also applied to test differences in fractional excretions of metabolites among the groups, where the fractional excretion of each metabolite is calculated by log2(metabolite in urine/metabolite in plasma). Finally we used PCA of significant metabolites to visually assess group differences.

The metabolite signature for type 2 diabetes was defined by the set of metabolites that met a false discovery rate (FDR) value of less than 5% followed by post-hoc Tukey analysis showing significant differences between the T2D group when compared to both OB and NW groups at p < 0.05. The metabolite signature for obesity was defined as the set showing significant differences between the OB and T2D group when compared to NW subjects. Pathways were identified by comparing the metabolites identified in each signature to published pathways in the Human Metabolome Database (hmdb.ca) or Kyoto Encyclopedia of Genes and Genomes (genome.jp/kegg/pathway) as well as pathway analysis using MetaboAnalyst (metaboanalyist.ca).

We used the R programming language for most of the statistical analyses including Spearman correlation analyses between metabolite levels versus clinical variables and aforementioned ANOVA and PCA. Fractional excretion was calculated as (urine metabolite/plasma metabolite)/(urine creatinine/serum creatinine)*100%.

## Results

### Subject characteristics and clinical data

Urine and plasma samples were provided by 90 adolescents (n = 30 in each of the 3 groups). Demographic and clinical lab data are shown in Table 1. The average age of each group was 15 ± 0.3 years old. There was an equal distribution of males and females in the OB and NW groups. Subjects were also matched for ethnicity and Tanner staging. At least two-thirds of each group consisted of subjects with Hispanic ethnicity, a reflection of the demographic makeup of Southern California.

**Table 1:** Subject characteristics and clinical data.

						Bonferroni *P* value		
		NW	OB	T2D	*P*	NW vs. OB	NW vs. T2D	OB vs. T2D
Characteristic					* *			
n		30	30	30				
Age (years)		15.7 ± 0.3	15.1 ± 0.3	15.5 ± 0.3	NS			
Sex								
	Male	14 (47%)	15 (50%)	19 (63%)	NS			
	Female	16 (53%)	15 (50%)	11 (37%)				
Race								
	White	11 (37%)	2 (7%)	3 (10%)	0.04			
	Black	2 (7%)	2 (7%)	2 (7%)				
	Hispanic	13 (43%)	19 (63%)	18 (60%)				
	Asian	0 (0%)	4 (13%)	1 (3%)				
	Native Hawaiian	0 (0%)	1 (3%)	1 (3%)				
	Other	4 (13%)	2 (7%)	5 (17%)				
Ethnicity								
	Hispanic	22 (73%)	23 (77%)	26 (87%)	NS			
	Non-Hispanic	8 (27%)	7 (23%)	4 (13%)				
Tanner stage								
	III	2 (7%)	2 (7%)	0 (0%)	NS			
	IV	6 (20%)	4 (13%)	4 (13%				
	V	22 (73%)	24 (80%)	26 (87%)				
BMI		21.2 ± 0.5	38.2 ± 1.2	38.6 ± 1.3	<0.0001	<0.0001	<0.0001	NS
BMI Z-score		0.13 ± 0.12	2.42 ± 0.06	2.46 ± 0.08	<0.0001	<0.0001	<0.0001	NS
Fasting								
	Glucose (mg/dL)	89 ± 1	90 ± 2	179 ± 16	<0.0001	NS	<0.0001	<0.0001
	Insulin (mU/mL)	9.2 ± 0.9	37.4 ± 5.4	37.6 ± 5.8	<0.0001	<0.0001	<0.001	NS
	C-peptide (ng/mL)	1.54 ± 0.09	4 ± 0.35	4.33 ± 0.37	<0.0001	<0.0001	<0.001	NS
	HOMA-IR	2.0 ± 0.2	8.3 ± 1.1	15.0 ± 2.7	<0.0001	<0.01	<0.0001	<0.01
	HbA1C (%)	5.3 ± 0.1	5.6 ± 0.1	8.5 ± 0.5	<0.0001	NS	<0.0001	<0.0001
	HbA1C (mmol/mmol)	33.5 ± 0.7	37.7 ± 0.7	69.4 ± 5.0	<0.0001	NS	<0.0001	<0.0001
	Cholesterol (mg/dL)	154 ± 6	166 ± 4	176 ± 8	0.05	NS	<0.05	NS
	Triglycerides (mg/dL)	74 ± 6	156 ± 12	164 ± 16	<0.0001	<0.0001	<0.0001	NS
	HDL (mg/dL)	49 ± 2	35 ± 2	38 ± 2	<0.0001	<0.0001	<0.001	NS
	LDL (mg/dL)	90 ± 5	100 ± 3	106 ± 6	NS			
Urine microalbumin (mg/g)		9.9 ± 2.3	16.3 ± 4.8	30.3± 8.7	NS			
eGFR (mL/min/1.73 m^2^)		98.6 ± 2.9	101.9 ± 3.1	112.9 ± 3.4	<0.01	NS	<0.01	<0.05
AST (U/L)		31 ± 2	34 ± 4	48 ± 6	<0.01	NS	<0.05	NS
ALT (U/L)		28 ± 2	49 ± 8	68 ± 11	<0.01	NS	<0.01	NS
Diabetes medications								
	Metformin only	0	2 (7%)	15 (50%)	n/a			
	Insulin only	0	0	2 (7%)				
	Metformin and insulin	0	0	10 (33%)				
	Other	0	0	3 (10%)				
Diabetes duration (months)		n/a	n/a	10.8 ± 1.6	n/a			
Fam Hx type 2 diabetes		18 (60%)	24 (80%)	30 (100%)	<0.01	NS	<0.01	NS
Maternal gestational DM		1 (3%)	4 (13%)	14 (47%)	<0.0001	NS	<0.0001	<0.01
Mom overweight or obese		9 (30%)	12 (40%)	22 (73%)	<0.001	NS	<0.01	<0.05

Data are mean ± SEM or n (%). Overall *P*-values were based on ANOVA.

Abbreviations: DM, diabetes; Fam Hx, family history; n/a, not applicable; NS, not significant.

As expected, BMI, BMI z-score, insulin, and C-peptide were significantly higher in the OB and T2D groups versus NW controls. Insulin and C-peptide values were similar among OB and T2D groups. Fasting glucose and HbA1C were similar among OB and NW groups and significantly increased in the T2D group as expected. HOMA-IR, a measure of insulin resistance, was lowest in the NW group and highest in the T2D group. Both OB and T2D groups showed evidence of dyslipidemia with increased triglyceride and decreased HDL levels. All groups had a normal average eGFR, but the eGFR was lowest in the NW group and highest in T2D, likely reflecting glomerular hyperfiltration in some diabetic subjects. [21] The average duration of diabetes was 10.8 ± 0.46 months, with a range of 1–28 months, and average HbA1C of 8.5% ± 0.5%. Of the T2D group, 33% were on both metformin and insulin, 55% were on metformin alone, 7% were on insulin alone, and 3% were on another T2D drug. A few OB subjects were on metformin for the treatment of polycystic ovary syndrome.

### A distinct urine metabolomic signature for type 2 diabetes in youth

**Fig 1:**
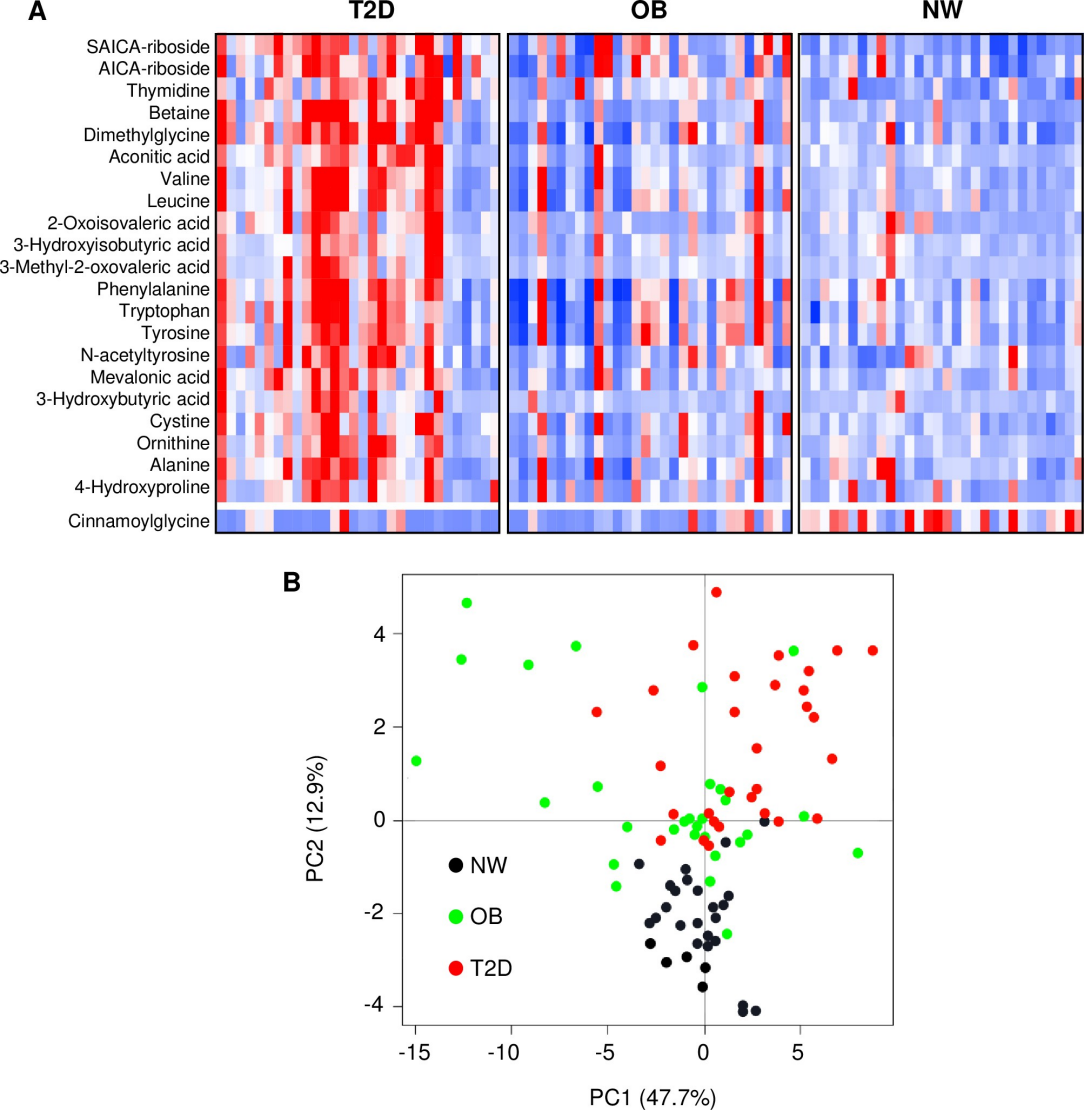
A distinct urine metabolomic signature for youth with type 2 diabetes. A) Heatmap shows relative urinary concentrations of the metabolites in the signature for adolescents with type 2 diabetes. The T2D signature consists of metabolites that met a 5% FDR cutoff with post-hoc Tukey T2D>NW and OB>NW, or T2D<NW and T2D<OB, *p*<0.05. Metabolites were grouped and listed by functional category. Blue boxes indicate metabolites with lower concentrations and red boxes indicate metabolites with higher concentrations. B) Principal components analysis reveals separation of diabetic youth from healthy and obese, non-diabetic controls when the 22 metabolites in the T2D signature were applied to all subjects. The figure shows the plot of principal component 1 (x-axis) versus principal component 2 (y-axis). Black circles represent NW control subjects, green circles represent OB subjects, and red circles represent T2D subjects.

In order to generate a urine metabolome signature for type 2 diabetes, we identified metabolites that met a false discovery rate (FDR) value of less than 5% (q-value < 0.05) using the BH method followed by post-hoc Tukey analysis showing significant differences between the T2D group when compared to both OB and NW groups at p < 0.05. There were 22 metabolites (excluding glucose) uniquely associated with our T2D subjects (Fig 1, S3 Table). Interestingly, the urine metabolite most significantly increased in T2D subjects was the purine intermediate succinylaminoimidazole carboxamide riboside (SAICA-riboside) followed by betaine metabolites (betaine and dimethylglycine).

**Fig 2:**
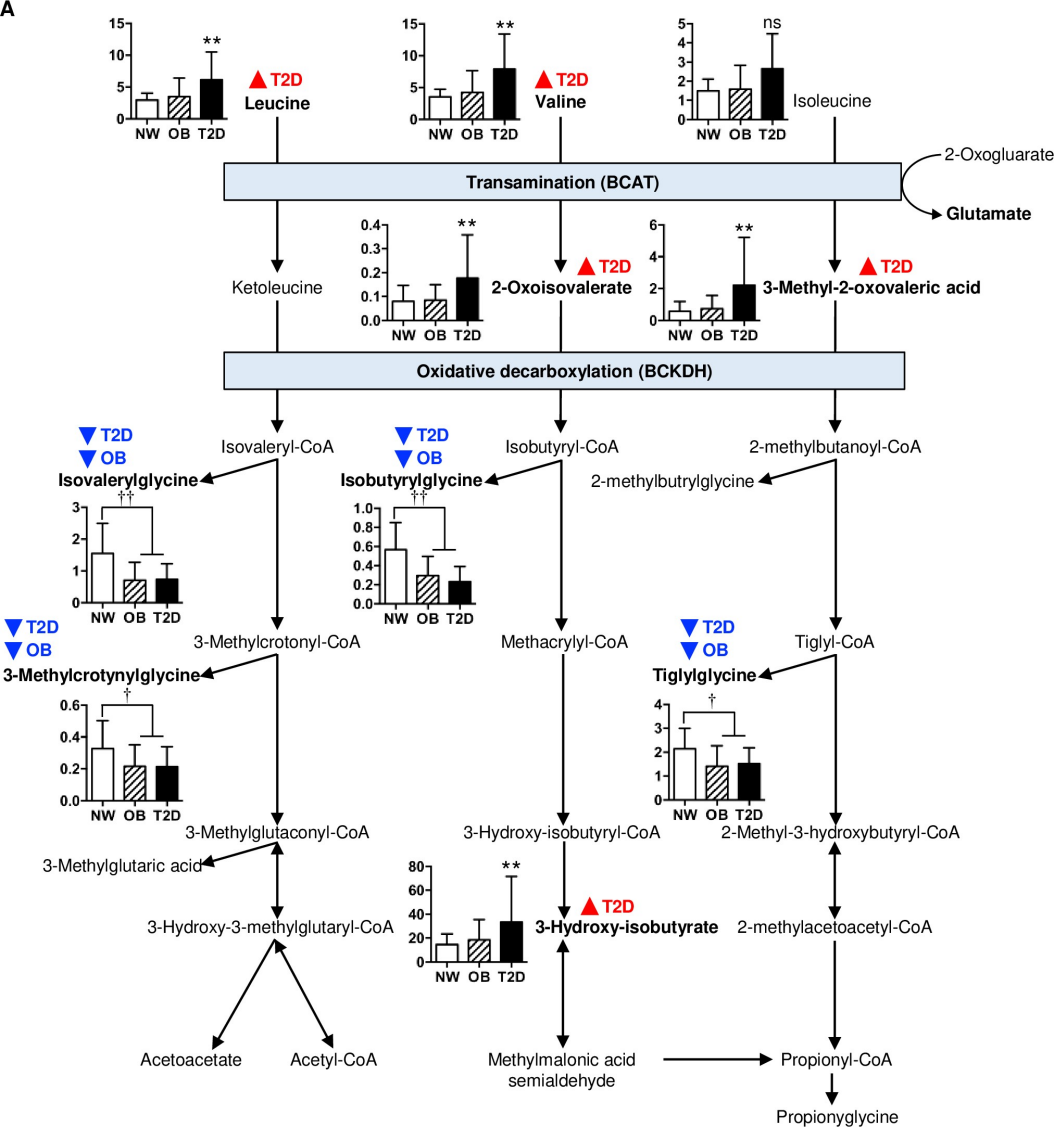
Urinary BCAA metabolites in NW, OB and T2D subjects. Urinary BCAAs and their immediate catabolites were increased in diabetic youth, but BCAA acylglycine derivatives were decreased in youth with obesity regardless of diabetes status. BCAA degradation pathways are shown. Red triangle symbols with T2D indicate post-hoc Tukey T2D>OB and T2D>NW. Blue triangle symbols with T2D indicate post-hoc Tukey T2D<NW, and blue triangle symbols with OB indicate post-hoc Tukey OB<NW. Bar charts show mean urinary metabolite concentrations ± SEM expressed as mmol/mmol creatinine: white bars, NW; hatched bars, OB; solid bars, T2D. **p<0.01 T2D vs. NW and T2D vs. OB, †p<0.05 compared to NW, ††p<0.01 compared to NW.

The largest set of urine metabolites represented in the T2D signature were the branched chain amino acids (valine and leucine) and their direct catabolic derivatives (2-oxoisovaleric acid, 3-methyl-2-oxovaleric acid, 3-hydroxyisobutyrate) which were all significantly increased in T2D subjects compared to their OB and NW counterparts (Fig 2), suggesting increased BCAA oxidative flux in diabetic youth. [22] Aromatic amino acids (phenylalanine, tyrosine and tryptophan) were also increased in the T2D group. Although we observed a general trend for higher values in OB vs. NW subjects for these urine metabolites, their differences did not reach statistical significance.

Principal components analysis of the 22 urine metabolites in our T2D signature demonstrated that the first two components separated the groups reasonably well (Fig 1B). The normal-weight subjects were isolated on the negative vertical axis. The obese subjects with and without diabetes co-migrated along the positive vertical axis. However, most of the diabetic subjects were located on the positive horizontal axis, in contrast to obese, non-diabetic subjects which tended to migrate on the negative horizontal axis.

### Metabolites associated with type 2 diabetes and obesity differ between urine and plasma

**Fig 3:**
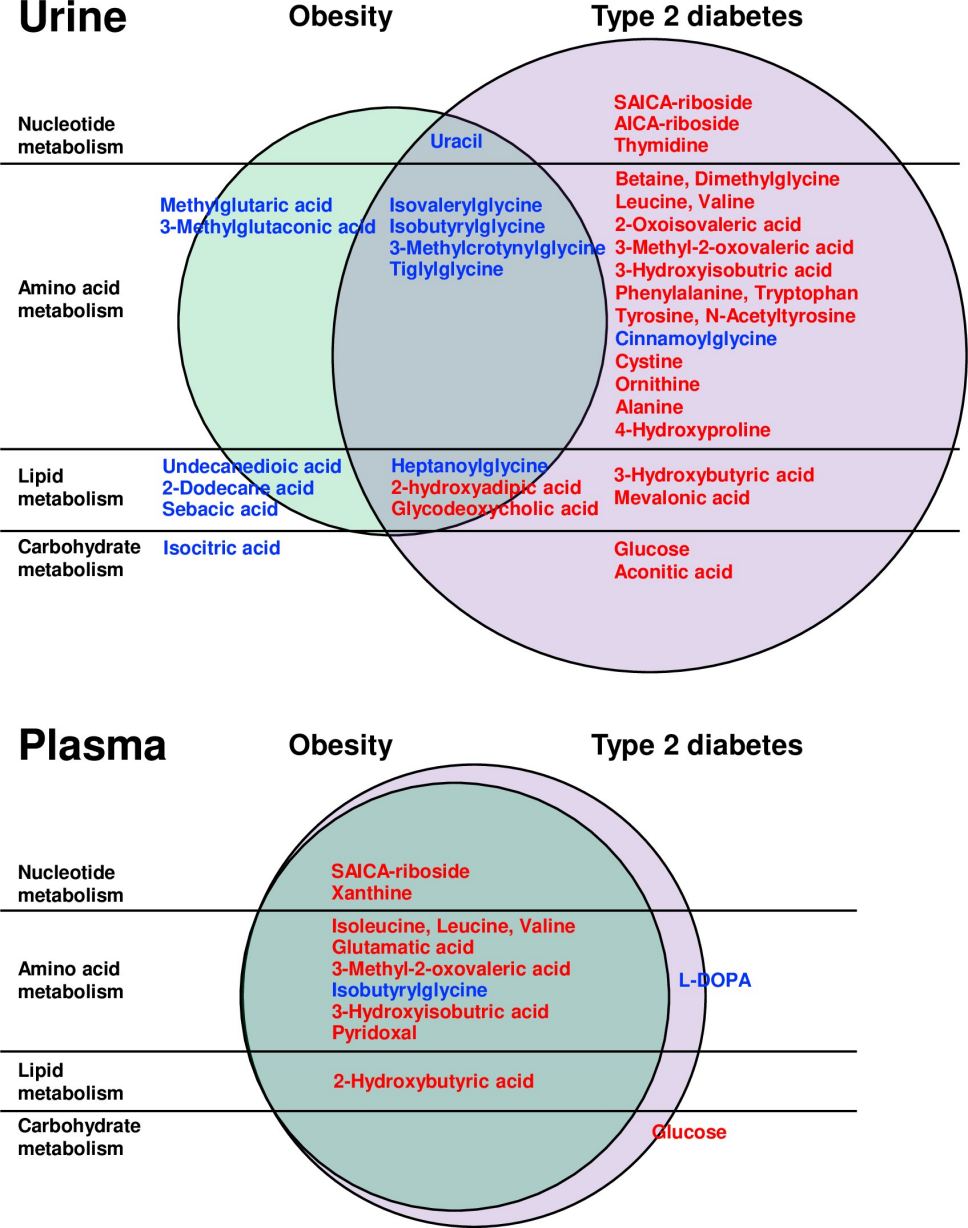
Differences in metabolite profiling between urine and plasma in youth with obesity and type 2 diabetes. The upper Venn diagram shows urine metabolites that were unique to the T2D signature in the right column (red font indicating T2D>OB and T2D>NW), blue font indicating T2D<OB and T2D<NW) or to the OB signature in the left column (red font indicating OB>TD and OB>NW, blue font indicating OB<T2D and OB<NW). Metabolites in the middle column represent metabolites altered in both OB and T2D groups compared to NW controls (red font indicating OB>NW andT2D>NW, blue font indicating OB<NW and T2D<NW). In contrast, the lower Venn diagram shows that only 2 plasma metabolites were unique to T2D subjects in the right column (red font indicating T2D>OB and T2D>NW), blue font indicating T2D<OB and T2D<NW), and no metabolites were unique to OB subjects alone. Instead, plasma metabolites in the left column were altered in both T2D and OB groups (red font indicating OB>NW and T2D>NW, blue font indicating OB<NW and T2D<NW), denoting that they were altered in obese subjects regardless of their diabetes status.

Although urine BCAAs and their catabolites were significantly higher in T2D subjects compared to OB or NW groups, we did not observe the same pattern in the fasting plasma metabolome. The plasma concentrations of BCAAs (valine, leucine and isoleucine) were higher in both T2D and OB groups when compared to NW controls, but not statistically different between the diabetic and non-diabetic obese subjects (Fig 3, S1 and S2 Figs and S4 Table). These data suggest that urine BCAAs and their intermediates serve as a more specific biomarker for T2D, whereas plasma BCAAs associate with the obese, insulin resistant state independent of diabetes status.

Plasma glutamate levels were increased (S4 Table, S1 Fig) and plasma 2-oxoglutarate/glutamate ratios were markedly lower in both the T2D and OB groups (S3 Fig), possibly arising from the conversion of 2-oxoglutarate to glutamate associated with the first step of BCAA degradation by branched-chain amino acid transaminase (BCAT). We also observed higher levels of plasma 2-hydroxybutyrate in T2D and OB subjects. These findings are similar to those previously demonstrated in insulin resistant adults. [23, 24]

We next examined which fasting plasma analytes (excluding glucose) that were uniquely associated with our T2D subjects by which metabolites met an FDR value of less than 5% using the BH step-down approach followed by post-hoc Tukey analysis showing significant differences between T2D subjects compared to both OB and NW groups at p < 0.05 (S2 Fig, S4 Table). The only plasma metabolite identified by this method was L-DOPA which was reduced in T2D subjects. When we measured the plasma ratio of tyrosine to L-DOPA as a proxy for tyrosine hydroxylase activity, we found lower enzyme activity in T2D subjects (S3 Fig). This metabolite was not identified in the urine T2D signature.

### Urine excretion of aconitic acid, betaine and thymidine is increased in adolescents with type 2 diabetes

To examine possible mechanisms that could contribute to the higher urine concentrations of these metabolites in our diabetic subjects, we analyzed their fractional excretion. We found a substantial increase in the fractional excretion of aconitic acid, 3-methyl-2-oxovaleric acid and betaine in diabetic adolescents which could account for their inclusion in the urine T2D signature (S4 Fig). Increased fractional excretion of betaine has been previously described in adults with type 2 diabetes independent of microalbuminuria. [25] Interestingly, the fractional excretion of L-DOPA was significantly higher in the T2D group, while the plasma concentrations of this metabolite were significantly lower in the same subjects. There were no changes in the fractional excretion of SAICA-riboside, the urine metabolite shown to be increased in our diabetic youth with the greatest statistical difference, indicating that alterations in fractional excretion did not account for all urine metabolite differences between groups.

### Urine acylglycines derived from BCAAs are reduced in obese subjects with and without type 2 diabetes

Although urine BCAA intermediates were increased in T2D subjects, the concentrations of urine acylglycines derived from these BCAA metabolites showed the opposite pattern, being significantly lower in both T2D and OB groups (Fig 2 and S5 Fig). These acylglycine species included those derived from valine (isobutrylglycine), leucine (isovalerylglycine, 3-methylcrotonylglycine), and isoleucine (tigylglycine). Lower urine acylglycine concentrations in T2D and OB subjects could reflect decreased activity of glycine-N-acyltransferase (GLYAT) to conjugate glycine with acyl-CoA substrates due to mitochondrial dysfunction in the liver or kidney. [26] Urine concentrations of cinnamoylglycine and heptanoylglycine, two other GLYAT-transformed products, were also lower in T2D subjects.

### Metabolites associated with clinical markers of insulin resistance and diabetes

We next identified which urine metabolites correlated with clinical markers of type 2 diabetes in our study population using Spearman rank correlation (Table 2). Interestingly, urinary valine and leucine were highly correlated with fasting glucose or HbA1c. In contrast, urinary acylglyine BCAA derivatives (isobutyrylglycine and isovalerylglycine) were highly correlated with insulin resistance as measured by HOMA-IR. Of note, the nucleic acid metabolites, thymidine and SAICA-riboside, were the only analytes significantly associated with both hyperglycemia and insulin resistance.

**Table 2:** Association of urinary and plasma metabolites with clinical variables of insulin resistance and type 2 diabetes.

			Fasting glucose		HbA1c		HOMA-IR		eGFR	
		* *	*r*	*P-value*	*r*	*P-value*	*r*	*P-value*	*r*	*P-value*	
**Urine**		* *	* *	* *	* *	* *	* *	* *	* *	* *	
	BCAA metabolism										
		Valine	0.41	0.027	0.51	<0.001			0.42	0.018	
		Leucine			0.46	0.003					
		Isobutyrylglycine					-0.59	<0.001			
		Isovalerylglycine					-0.57	<0.001			
		3-methyl-2-oxovalerate							0.40	0.035	
	Choline pathway										
		Betaine	0.42	0.030	0.56	<0.001			0.45	0.006	
		Dimethylglycine	0.43	0.016	0.43	0.013					
	Nucleic acid metabolism										
		Thymidine	0.46	0.003	0.52	<0.001	0.50	0.001			
		Uracil					-0.50	0.001			
		Saicar			0.47	0.002	0.45	0.012			
	Lipid metabolism										
		Mevalonate					0.43	0.038	0.40	0.037	
		3-hydroxybutyrate	0.42	0.034	0.52	<0.001					
		Heptanoylglycine			-0.44	0.011					
	TCA cycle										
		Aconitate	0.40	0.047	0.49	0.001					
	Aromatic amino acids										
		Phenylalanine	0.41	0.030	0.42	0.022					
		Tyosine	0.42	0.024	0.41	0.030					
		Tryptophan			0.45	0.006					
	Other amino acids										
		Alanine							0.44	0.005	
		Cystine	0.46	0.004	0.43	0.018			0.40	0.044	
		Ornithine	0.44	0.007	0.41	0.033			0.42	0.018	
		4-hydroxyproline			0.43	0.015			0.43	0.010	
**Plasma**											
	BCAA metabolism										
		Valine			0.63	0.002					
		Leucine			0.56	0.002					
		3-methyl-2-oxovalerate			0.63	0.037					

*P*-values represent Bonferroni-adjusted Spearman Rank correlation *P*-values.

Highly significant associations with clinical markers for glucose metabolism were found in other urinary metabolites included in the T2D metabolite signature. Nucleic acid metabolites (thymidine, uracil and SAICA-riboside), betaine metabolites (betaine and dimethyglycine), lipid metabolites (3-hydroxybutyrate and heptanoylglycine), aromatic amino acids (phenylalanine, tyrosine and tryptophan) and the TCA metabolite aconitate were all strongly linked to hyperglycemia as measured by fasting glucose and HbA1c. When plasma metabolites were subjected to a similar analysis, BCAA metabolites (valine, leucine, isoleucine and 3-methyl-2-oxovalerate) were found to correlate with HbA1c, but no other associations were identified.

We also correlated metabolites to eGFR as our diabetic subjects had significantly higher filtration rates compared to their non-diabetic counterparts. Urinary betaine and its product dimethylglycine were strongly associated with eGFR. Other urine metabolites in this category included alanine, 4-hydroxyproline, ornithine, mevalonic acid, and the BCAA metabolites valine and 3-methyl-2-oxovalerate, suggesting that these metabolites may serve as a biomarker for early diabetic renal injury prior to the development of microalbuminuria.

## Discussion

Insulin resistance and obesity are major risk factors for the development of type 2 diabetes, cardiovascular disease, and premature death. Prior metabolomic studies in obese youth without diabetes showed elevated fasting serum concentrations of BCAAs and 3-hydroxybutyrate, similar to adults. [8, 9, 27] In contrast, studies of subjects with type 2 diabetes yielded contradictory results; elevated fasting serum concentrations of BCAAs and aromatic amino acids were observed in diabetic adults [28], but were reported to be lower in diabetic youth. [29]

The primary focus of this study was to determine whether urine and plasma metabolites were uniquely associated with early-stage type 2 diabetes in adolescents when compared to non-diabetic obese and normal-weight control subjects (Figs 1 and 3 and S6 Fig). We were able to identify a subset of 22 urine metabolites that differed significantly in T2D youth when compared to their OB and NW counterparts.

SAICA-riboside was the metabolite with the highest statistical significance in the T2D urine signature. Urinary SAICA-riboside and thymidine also strongly correlated with clinical markers of hyperglycemia and insulin resistance. Insulin resistance and other states of oxidative stress states are postulated to reduce glucose oxidation and increase glucose flux through the pentose phosphate pathway to produce NADPH and to synthesize nucleotides [30]. SAICAR, an intermediate in *de novo* purine nucleotide synthesis, can arise from ribose 5-phosphate generated from this pathway, and can be dephosphorylated to SAICA-riboside. Furthermore, cleavage of SAICAR to AICAR and fumarate, and cleavage of S-AMP to AMP and fumarate during *de novo* synthesis of AMP are both catalyzed by a single enzyme, adenylosuccinate lyase (ADSL). The ADSL reaction is also a source of fumarate, an anaplerotic metabolite replenishing the TCA cycle and inducing ATP production. Prior work has shown the inhibition of ADSL in an insulinoma cell line lowered S-AMP, an insulin secretagogue, to impair glucose-stimulated insulin secretion. [31] In our study, plasma concentrations and the fractional excretion of SAICA-riboside did not differ between groups. Moreover, plasma and urine concentrations of succinyladenosine, which would be lowered in this paradigm, were also unchanged. However, urine fumarate was increased in T2D youth. Another explanation for the increased levels of SAICA-riboside could be that elevated fumarate inhibits the conversion of SAICAR to AICAR. As AICAR is known to stimulate AMPK, a reduction in AICAR would be linked to reduced AMPK, which is a feature we and others have consistently demonstrated in the diabetic kidney. [32] The elevation in fumarate is likely linked to reduced kidney fumarate hydratase levels as seen in our recent study in mice and humans with diabetic kidney disease. [33]

Interestingly, betaine and nucleic acid metabolite concentrations were significantly increased in T2D youth. In addition, six of the 22 metabolites represented BCAAs and higher plasma concentrations of BCAAs and branched-chain α-ketoacids derived from BCAAs [23] as well as C3 and C5 acylcarnitines derived from BCAA oxidation products [6], reflecting altered BCAA catabolic flux. In contrast to prior measurements in diabetic youth [29], our study shows that fasting plasma BCAA concentrations and several related metabolites (3-methyl-2-oxovalerate, 3-hydroxyisobutyrate and glutamate) were higher in obese adolescents regardless of diabetes status, indicating that circulating BCAA levels are elevated in obese and diabetic youth, similar to older adults. The plasma glutamine/glutamate ratio was lower in the T2D and OB groups, also similar to adults [24], and consistent with increased oxidative flux. Of note, although plasma levels of BCAAs and their oxidation products were similar between OB and T2D groups, there was a marked increase in the urine concentrations of BCAAs and their catabolites in T2D but not OB subjects when compared to NW controls. This difference in metabolite pattern between urine and plasma could be explained in part by differences in fractional excretion.

Surprisingly, few plasma metabolites were uniquely associated with the T2D group. However fasting plasma L-DOPA levels were markedly lower in T2D subjects, accompanied by a higher plasma tyrosine/L-DOPA ratio suggesting reduced activity of tyrosine hydroxylase in diabetes. Intrarenal dopamine in the kidney has been noted to be reduced in diabetic mice and may contribute to progressive renal disease. [34] Our demonstration of reduced plasma L-DOPA may indicate a biomarker for those with progressive diabetic nephropathy.

In summary, adolescent subjects with early-stage diabetes without major co-morbid disease, as seen in diabetic adults, have evidence of altered purine nucleotide metabolism, betaine metabolism and oxidative BCAA flux. Our results also showed that metabolite profiles differed between urine and plasma. In addition, urinary metabolites in the diabetes signature correlated with different clinical variables: betaine, BCAAs and aromatic amino acids were tightly correlated with hyperglycemia; BCAA acylglycine derivatives were linked to insulin resistance; and the nucleic acid metabolites, thymidine and SAICA-riboside were the only analytes associated with both hyperglycemia and insulin resistance. Betaine, 4-hydroxyproline and other amino acids correlated with renal hyperfiltration and potentially early nephropathy. Future longitudinal studies will be warranted to determine whether these metabolites serve as useful biomarkers that predict the development of diabetes and its complications in children.

## Supporting information

S1 Table List of all standards used in this study. (PDF)

S2 Table List of all precursor-product ion transitions for negative and positive ionization modes. (XLSX)

S3 Table Comparison of urine metabolites in the signature for type 2 diabetes and the signature for obesity with or without diabetes. Urine metabolite concentrations were measured as mmol/mmol creat and log2-transformed for subsequent analysis. Data here are reported as mean and 95% confidence intervals after inverse log transformation. T2D signature: Post-hoc Tukey T2D>OB and T2D>NW, or T2D<NW and T2D<OB Obesity signature: Post-hoc Tukey T2D>NW and OB>NW, or T2D<NW and OB<NW.
(PDF)

S4 Table Comparison of plasma metabolites in the signature for type 2 diabetes and the signature for obesity with or without diabetes. Plasma metabolite concentrations were measured as mM and log2-transformed for subsequent analysis. Data here are reported are mean and 95% confidence intervals after inverse log transformation. T2D signature: Post-hoc Tukey T2D>OB and T2D>NW, or T2D<NW and T2D<OB Obesity signature: Post-hoc Tukey T2D>NW and OB>NW, or T2D<NW and OB<NW
(PDF)

S1 Fig Comparison of urine and plasma BCAA metabolites associated with type 2 diabetes or obesity A) Urinary BCAAs and several of their degradation products are increased in T2D compared to OB and NW controls. In contrast, plasma BCAAs and their catabolites are increased in both T2D and OB groups compared to NW controls. B) BCAA degradation pathways are shown. Red triangle symbols with T2D indicate post-hoc Tukey T2D>NW, and red triangle symbols with OB indicate post-hoc Tukey OB>NW. Blue triangle symbols with T2D indicate post-hoc Tukey T2D<NW, and blue triangle symbols with OB indicate post-hoc Tukey OB<NW.
(TIFF)

S2 Fig Plasma signatures associated with type 2 diabetes or obesity. Signatures consists of metabolites that met a 5% FDR cutoff.Type 2 diabetes signature: Post-hoc Tukey T2D>OB and T2D>NW, or T2D<NW and T2D<OB Obesity signature: Post-hoc Tukey T2D>NW and OB>NW, or T2D<NW and OB<NW
(TIFF)

S3 Fig Metabolite ratios used to infer enzyme activity A) 2-Oxoglutarate to glutamate ratio: branched-chain amino acid transaminase activity. B) Glutamate to glutamine ratio: glutamine synthetase activity. C) Tyrosine to L-DOPA ratio: tyrosine hydroxylase activity. **p<0.01 vs. NW, ***p<0.001 vs. NW, ****p<0.0001 vs NW, †p<0.05 vs. T2D.
(TIFF)

S4 Fig Metabolites with increased fraction excretion in T2D. Post-hoc Tukey T2D>NW and T2D>OB at p≤0.05.
(TIFF)

S5 Fig Urine metabolites in the signature for obesity with or without diabetes. Obesity signature: Post-hoc Tukey T2D>NW and OB>NW, or T2D<NW and T2D<OB.
(TIFF)

S6 Fig Pathway diagram showing the relationship of urine metabolites altered in obesity and type 2 diabetes. Red triangle symbols with T2D indicate post-hoc Tukey T2D>NW, and red triangle symbols with OB indicate post-hoc Tukey OB>NW. Blue triangle symbols with T2D indicate post-hoc Tukey T2D<NW, and blue triangle symbols with OB indicate post-hoc Tukey OB<NW.
(TIFF)

S1 Data (XLSX)

S2 Data (XLSX)
