# Supplementary material for: Identification of pathognomonic purine synthesis biomarkers by metabolomic profiling of adolescents with obesity and type 2 diabetes
Source: PLoS One. 2020 Jun 26;15(6):e0234970. doi: 10.1371/journal.pone.0234970 (PMC7319336; doi:10.1371/journal.pone.0234970)
Supplement: S1 Table — (PDF) [file pone.0234970.s001.pdf]

Organic Acids

Chemical	Company	Reference
2-hydroxy-3-methylvaleric	Sigma Aldrich	H2752
2-hydroxyadipic	UAM	OA 101-OH SC
2-hydroxybutyric	Sigma Aldrich	H1253
2-hydroxybutyric d3	CDN Isotopes	D-7002
2-hydroxydecanedioic	UAM	OA 123 SC
2-hydroxyglutaric	Sigma Aldrich	H8253
2-hydroxyisocaproic	Sigma Aldrich	H9251
2-hydroxyisovaleric	Sigma Aldrich	55454
2-hydroxyphenylacetic acid	Sigma Aldrich	H4980
2-Methyl-3-hydroxybutyric acid	UAM	OA 105 SC
2-methylacetoacetic	Sigma Aldrich	537365
2-methylcitric	UAM	OA 102
2-octenedioic	UAM	OA 130 SC
2-oxo-3-methylvaleric	Sigma Aldrich	K7125
2-oxoadipic	Sigma Aldrich	K5875
2-oxobutyric	Sigma Aldrich	K0875
2-oxoglutaric	Sigma Aldrich	K2000
2-oxoisocaproic	Sigma Aldrich	68255
2-oxoisocaproic d3	CDN Isotopes	D-2914
2-oxoisovaleric	Sigma Aldrich	198994
2-oxoisovaleric 13C	Cambridge Isotopes	CDLM-8100
3-hydroxy-2-ethylpropionic	UAM	OA 104 SC
3-hydroxy-3-methylglutaric	Sigma Aldrich	H1752
3-hydroxy-3-methylglutaric d3	CDN Isotopes	MD3772
3-hydroxyadipic	UAM	OA 106 SC
3-hydroxybutyric	Sigma Aldrich	H6501
3-hydroxybutyric 13C	CDN Isotopes	C3320
3-hydroxybutyric d3	CDN Isotopes	MD2480
3-Hydroxydecanedioic acid	UAM	OA 120 SC
3-hydroxyglutaric	UAM	OA 107
3-hydroxyglutaric d5	CDN Isotopes	D6391
3-hydroxyisobutyric	UAM	OA 108 SC
3-hydroxyisovaleric	UAM	OA 109
3-hydroxyisovaleric d8	Cambridge Isotopes	DLM-10603
3-hydroxypropionic	Sigma Aldrich	H55006
3-hydroxyvaleric	UAM	OA 111
3-methyladipic	Sigma Aldrich	M27409
3-methylcrotonylglycine	UAM	AG 110 SC
3-methylglutaconic	UAM	OA 119
3-methylglutaric acid	Fluka Chemika	66990
3-methylglutaric acid d4	CDN Isotopes	D-6723
4-hydroxy-3-methoxyphenylglycol sulfate	Sigma Aldrich	H8759
3-oxoadipic	Sigma Aldrich	K-0500
3-oxovaleric	UAM	OA 131 SC
4-hydroxybutyric	Sigma Aldrich	H3635
4-Hydroxycyclohexylacetate	UAM	OA 113 SC
4-hydroxyisovaleric	UAM	OA 114 SC
4-hydroxyphenylacetic acid	Sigma Aldrich	H4377
4-hydroxyphenylacetic acid d2	CDN Isotopes	MD2146
4-hydroxyphenyllactic	Sigma Aldrich	H3253
4-hydroxyphenylpyruvic	Sigma Aldrich	H4880
4-pyridoxic	Sigma Aldrich	P9630
5-hydroxyhexanoic	UAM	OA 115
5-hydroxyindole-3-acetic	Sigma Aldrich	H0355
5-hydroxyindole-2-carboxylic	Sigma Aldrich	143510
5-Methoxytryptophol	Fluka Chemika	M4126
5-oxoproline	Fluka Chemika	83170
7-hydroxyoctanoic	UAM	OA 129 SC
acetoacetic	Sigma Aldrich	A8509
aconitic	Sigma Aldrich	A3412
adipic	Sigma Aldrich	A5252
adipic 13C	Cambridge Isotopes	CLM-4723
ascorbic	Sigma Aldrich	A5960
ascorbic-2-sulfate	Sigma Aldrich	A9659
azelaic	Sigma Aldrich	246379

azelaic d14	CDN Isotopes	D-3280
benzoic	Sigma Aldrich	B3250
butyrylglycine	UAM	AG 103 SC
cinnamoylglycine	UAM	ukn
citric acid	Sigma Aldrich	251275
citric acid d4	CDN Isotopes	D3745
decanoic acid	Fluka Chemika	21409
decanoic d3	Cambridge Isotopes	DLM-2006
dehydroascorbic	Sigma Aldrich	261556
dodecanedioic	Sigma Aldrich	D-9630
ethylmalonic	Sigma Aldrich	102687
ethylmalonic d3	CDN Isotopes	D-5145
Fumaric	Sigma Aldrich	240745
fumaric d2	CDN Isotopes	D-1106
furan-2,5-dicarboxylic	UAM	OA 117
glutaconic	Fluka Chemika	49360
glutaric	Sigma Aldrich	G3407
glutaric d4	CDN Isotopes	MD1197
glyceric	Sigma Aldrich	G8766
glycolic	Sigma Aldrich	G8284
glycolic d2	Cambridge Isotopes	DLM-399
glyoxylic	Sigma Aldrich	G4502
hexanoylglycine	UAM	AG 105 SC
hexanoylglycine d2	CDN Isotopes	D-5992
hippuric	Sigma Aldrich	112003
hippuric d2	MSD Isotopes	MD-1707
homogentisic	Sigma Aldrich	H0751
homovanillic	Sigma Aldrich	H1252
homovanillic d3	Cambridge Isotopes	DLM-2738
indole-3-acetic	Sigma Aldrich	I5148
indole-3-butyric	Sigma Aldrich	I7512
indole-3-carboxaldehyde	Sigma Aldrich	129445
indole-3-carboxylic	Sigma Aldrich	284734
indole-3-lactic	Sigma Aldrich	I5508
indole-3-propionic	Sigma Aldrich	57400
indole-3-pyruvic	Sigma Aldrich	I7017
indolelactic	Sigma Aldrich	I2875
indoxylacetic	Sigma Aldrich	I3500
indoxyl-beta-glucuronide	Sigma Aldrich	I7638
isobutyrylglycine	UAM	AG 103 SC
isobutyrylglycine d7	CDN Isotopes	D-7636
isocitric	Sigma Aldrich	I-1252
isovalerylglycine	UAM	AG 107
kynurenic	Sigma Aldrich	K8625
lactic	Sigma Aldrich	L-1500
lactic 13C	isotech	T8362033
maleic	Sigma Aldrich	63180
malic	Sigma Aldrich	M6773
malic d3	CDN Isotopes	D-2122
malonic 13C	Cambridge Isotopes	CLM-1248
methylbutyrylglycine	UAM	AG 117 SC
methylbutyrylglycine d9	CDN Isotopes	D-6709
methylmalonic	Sigma Aldrich	M54058
methylmalonic d3	Cambridge Isotopes	DLM-387
methylsuccinic	Sigma Aldrich	415596
mevalonic	UAM	OA 342
N-acetyl-aspartic	Sigma Aldrich	920
N-acetylneuraminic	Sigma Aldrich	19023
N-acetyl-tyrosine	Calbiochem	11116
orotic	Sigma Aldrich	O1756
oxalic	Fluka Chemika	4621
oxalic 13C	Cambridge Isotopes	CLM-2002
p-acetamidophenyl ß-D-glucuronide	Sigma Aldrich	92564
pantothenic	Sigma Aldrich	P2250
phenylacetic	Sigma Aldrich	P4514
phenyllactic	Sigma Aldrich	P7251
phenylpyruvic	Sigma Aldrich	P8001
pimelic	Sigma Aldrich	P45001

pimelic d4/ heptanedioic d4	CDN Isotopes	D-2004
propionylglycine	UAM	AG 112 SC
pyruvic	Sigma Aldrich	P2256
pyruvic 13C	Cambridge Isotopes	CLM-3507
quinolinic	Sigma Aldrich	P63204
sebacic	Sigma Aldrich	28325
sebacic d4	CDN Isotopes	Md1065
suberic	Sigma Aldrich	S5200
suberic d4	CDN Isotopes	D-2008
suberylglycine	UAM	AG 113 SC
succinic	Sigma Aldrich	134384
succinic 13C	Cambridge Isotopes	CLM-1571
succinylacetone	Sigma Aldrich	D-1415
tetradecanedioic	Sigma Aldrich	D221201
tiglylglycine	UAM	AG 114
tiglylglycine d2	CDN Isotopes	D-6615
tridecanedioic	Sigma Aldrich	U601
undecanedioic	Sigma Aldrich	177962
uracil	Sigma Aldrich	U-0750
uracil 15N	Cambridge Isotopes	NLM-637
uric	Sigma Aldrich	U2875
uric 15N	Cambridge Isotopes	NLM-1697
valerylglycine	UAM	AG 115
vanylglycol (MHPG)	Sigma Aldrich	H1377
vanylmandelic	Sigma Aldrich	H0131
vanylmandelic d3	CDN Isotopes	D3919
xanthurenic	Calbiochem	68388

Amino Acids

1-methylhistidine[2]	Sigma Aldrich	67520
2-aminoadipic[1]	Sigma Aldrich	A7275
2-aminobutyric	Sigma Aldrich	162663
3-methylhistidine[2]	Sigma Aldrich	M9005
5-aminolevulinic	Sigma Aldrich	A3785
5-hydroxypipecolic	Calbiochem	396212
5-hydroxytryptophan	Sigma Aldrich	H8127
alanine[1]	Sigma Aldrich	5129
alanine d4	Cambridge Isotopes	DLM-1276
alpha amino gamma guanidinobutyric	Calbiochem	1548
anserine[1]	Wako	011-14463
arginine[2]	Sigma Aldrich	A5131
argininosuccinic	Sigma Aldrich	A5707
asparagine	Sigma Aldrich	A0884
asparagine 15N	Cambridge Isotopes	NLM-3286
aspartic	Sigma Aldrich	A9256
aspartic acid d3	Cambridge Isotopes	DLM-546
beta-alanine[1]	Sigma Aldrich	5160
cadaverine	Sigma Aldrich	33220
canavanine	Calbiochem	2090
carnosine[2]	Sigma Aldrich	2180
citrulline	MP Biomedical	101394
creatine	Sigma Aldrich	MET-250A
creatine	Calbiochem	2380
creatine d3	CDN Isotopes	D-1972
creatinine	Sigma Aldrich	C4255
creatinine d3	CDN Isotopes	D-3689
cystathionine[1]	Sigma Aldrich	C-3633
cysteine	Sigma Aldrich	30120
cysteine d2	Cambridge Isotopes	DLM-769
cysteinyl-glycine	Sigma Aldrich	C-0166
cystine[1]	Sigma Aldrich	C8755
cystine d4	Cambridge Isotopes	DLM-1000
dimethylarginine	Calbiochem	311201
dimethylglycine	Sigma Aldrich	D1156
4-Aminobutyric[2]	Calbiochem	1370
4-Aminobutyric d2	CDN Isotopes	D-6846

gamma-butyrobetaine	Sigma Aldrich	403245
glucosamine	Calbiochem	3462
glutamic[1]	Sigma Aldrich	G1251
glutamic d3	Cambridge Isotopes	DLM-3725
glutamine	Sigma Aldrich	49419
glutamine d5	Cambridge Isotopes	CLM-1826
glycine[1]	Sigma Aldrich	G-7126
glycine 2-13C-15N	Cambridge Isotopes	CNLM-508
glycylglycine	Sigma Aldrich	G-1002
glycylproline	Sigma Aldrich	G-3002
Guanidinoacetic	Sigma Aldrich	G11608
Guanidinoacetic d2	CDN Isotopes	D-6320
histidine[2]	Sigma Aldrich	H8125
histidine d3	CDN Isotopes	D-6679
homoarginine	Calbiochem	U382125
homocitrulline	Toronto Research	H590900
homocysteine	Sigma Aldrich	H-4628
homocystine	Sigma Aldrich	H-0501
homocystine d8	Cambridge Isotopes	DLM-3619
hydroxyproline[1]	Sigma Aldrich	H-6002
isoleucine[1]	Sigma Aldrich	I-2752
kynurenine	Sigma Aldrich	K8625
leucine[1]	Sigma Aldrich	L-8000
leucine d3	Cambridge Isotopes	DLM-1259
lysine[2]	Sigma Aldrich	L5626
lysine d4	Cambridge Isotopes	DLM-2640
methionine[1]	Sigma Aldrich	M-9625
methionine 13C d3	Cambridge Isotopes	CDLM-760
N6,N6,N6-Trimethyllysine	Sigma Aldrich	T1660
ornithine[2]	Sigma Aldrich	O-2375
ornithine-d2	Cambridge Isotopes	DLM-4261
phenylalanine[1]	Sigma Aldrich	P-2126
phenylalanine d5	Cambridge Isotopes	DLM-1258
pipecolic	Sigma Aldrich	P2519
pipecolic d9	Sigma Aldrich	P2520
proline[1]	Sigma Aldrich	81709
proline d7	Cambridge Isotopes	DLM-487
putrescine	Sigma Aldrich	51799
S-adenosylhomocysteine	Sigma Aldrich	A9384
S-adenosylmethionine	Sigma Aldrich	A7007
sarcosine[1]	Sigma Aldrich	S7672
serine[1]	Calbiochem	5650
serine d3	Cambridge Isotopes	DLM-582
spermidine	Calbiochem	56766
taurine[1]	Fluka	86329
taurine d4	CDN Isotopes	D-1971
threonine[1]	Sigma Aldrich	T-8625
threonine d2	CDN Isotopes	D-6362
trimethylamine N-oxide	Sigma Aldrich	317594
trimethylamine N-oxide d9	Cambridge Isotopes	DLM-603
tryptophan	Sigma Aldrich	T-0254
tryptophan d5	Cambridge Isotopes	DLM-1092
tyrosine[1]	Sigma Aldrich	T-3754
tyrosine d4	Cambridge Isotopes	DLM-451
valine[1]	Sigma Aldrich	V0500
valine d8	Cambridge Isotopes	DLM-311

[1] Also part fujifilm wako pure chemical pure amino acids mix AN-II (011-14483).
[2] Also part fujifilm wako pure chemical pure amino acids mix Type B (012-08643).

Purines and Pyrimidines

1-methyladenosine	Sigma Aldrich	47310-U
2′-O-methylcytidine	Sigma Aldrich	47310-U
2-thiocytidine	Sigma Aldrich	47310-U
3-aminoisobutyric	Sigma Aldrich	217794
3-methylcytidine	Sigma Aldrich	47310-U

5-methylcytidine	Sigma Aldrich	47310-U
5-methyluridine	Sigma Aldrich	47310-U
5'-deoxy-methylthioadenosine	Sigma Aldrich	D5011
7-methylguanine	Sigma Aldrich	67073
7-methylguanosine[3]	Sigma Aldrich	M0627
7-methylinosine	Sigma Aldrich	M8382
adenine	Sigma Aldrich	A9126
adenine 13C	Cambridge Isotopes	CLM-1654
adenosine	Calbiochem	116801
adenosine 13C	Cambridge Isotopes	CLM-7674
AICAr (5-amino-1-(B-D-ribofuranosyl)-imidazole-4-carboxamide)	Sigma Aldrich	A-8129
allantoin	Fluka	5670
caffeine	Sigma Aldrich	27600
cytidine[3]	Sigma Aldrich	C4654
deoxyadenosine	Sigma Aldrich	D7400
deoxycytidine	Sigma Aldrich	D8006
deoxyguanosine	Sigma Aldrich	D7145
deoxyinosine	Sigma Aldrich	D5287
deoxyuridine	Sigma Aldrich	D5412
dihydrouracil	Sigma Aldrich	D7628
guanine	Sigma Aldrich	51030
guanine 13C	Cambridge Isotopes	CLM-1019
guanosine[3]	Santa Cruz	SC-218575
guanosine 15N5	Cambridge Isotopes	NLM-3798
hypoxanthine	Sigma Aldrich	H9377
hypoxanthine d4	Cambridge Isotopes	DLM-2923
Inosine[3]	Sigma Aldrich	I4125
Inosine 15N4	Cambridge Isotopes	NLM-4264
orotidine	Sigma Aldrich	O-6129
paraxanthine	Sigma Aldrich	D5385
SAICAr (N-succinyl-5-aminoimidazole-4-carboxamide ribose)	Toronto Research	S688790
succinyladenosine	Toronto Research	S688825
theobromine	Sigma Aldrich	T4500
theophylline	Sigma Aldrich	T1633
thymidine	Fluka	89270
thymidine 13C	Cambridge Isotopes	CLM-3647
thymine	Sigma Aldrich	T0376
thymine d4	Cambridge Isotopes	DLM-1089
ureidopropionic	Sigma Aldrich	94295
uridine[3]	Sigma Aldrich	U6381
uridine d2	CDN Isotopes/MSD Iso	D-5291
xanthine	Sigma Aldrich	X2502
xanthine 15N	Cambridge Isotopes	NLM-1698
β-Pseudouridine	Sigma Aldrich	47310-U

3also part of Sigma Aldrich 47310-U

Acylcarnitines

carnitine	Sigma Aldrich	C0283
carnitine d3	CDN Isotopes	D-5069
acetylcarnitine (C2)	Sigma Aldrich	A-6706
acetylcarnitine d3 (C2)	AMC	n/a
propionylcarnitine (C3)	AMC	n/a
propionylcarnitine d5 (C3)	AMC	n/a
butyrylcarnitine (C4)	AMC	n/a
isobutyrylcarnitine (isoC4)	AMC	n/a
isobutyrylcarnitine d7 (isoC4)	AMC	n/a
2-methybutyrylcarnitine (C5)	AMC	n/a
valerylcarnitine (C5)	AMC	n/a
isovalerylcarnitine (isoC5)	AMC	n/a
isovalerylcarnitine d9 (isoC5)	AMC	n/a
hexanoylcarnitine (C6)	AMC	n/a
octanoylcarnitine (C8)	AMC	n/a
octanoylcarnitine d3 (C8)	AMC	n/a
decanoylcarnitine (C10)	AMC	n/a
lauroylcarnitine (C12) / dodecanoylcarnitine	AMC	n/a
myristoylcarnitine (C14)/ tetradecanoyl-L-carnitine	AMC	n/a

palmitoylcarnitine d3(C16)/ hexadecanoyl-L-carnitine	AMC	n/a
stearoylcarnitine (C18) / octadecanoylcarnitine	AMC	n/a

Sugars

glucose	Sigma Aldrich	G8270
glucose d2	Cambridge Isotopes	DLM-349
N-Acetylgalactosamine	Sigma Aldrich	A-4016

Vitamins

choline	Sigma Aldrich	C7017
5-methyltetrahydrofolic	Sigma Aldrich	M0132
9-cis-retinoic	Sigma Aldrich	R4643
alpha-tocopherol	Sigma Aldrich	47783
betaine	Sigma Aldrich	B2629
biopterin	Dr. Schircks	11.203
dihydrobiopterin	Dr. Schircks	11.206
dihydroneopterin	Dr. Schircks	11.306
folic	Sigma Aldrich	F7876
gamma-tocopherol	Sigma Aldrich	47785
neopterin	Dr. Schircks	11.303
pantothenic/ vitamin B5	Sigma Aldrich	P2250
pyridoxal	Sigma Aldrich	P9130
riboflavin/vitamin B2	Sigma Aldrich	R4500
tetrahydrofolic	MP Biomedicals	101131
thiamine/ vitamin B1	Sigma Aldrich	T4625
trans-retinoic Acid	Calbiochem	554720

Neurotransmitters

3,4-dihydroxyphenylacetic acid (DOPAC) ring-d3 d2	Cambridge Isotopes	DLM-2499
acetylcholine	Sigma Aldrich	A6625
DOPA/3,4-dihydroxyphenylalanine	Sigma Aldrich	D9628
dopamine d3	Cambridge Isotopes	DLM-2181
dopamine/ 3-hydroxytyramine	Sigma Aldrich	H8502
octopamine	Sigma Aldrich	68631
norepinephrine	Sigma Aldrich	A7256
serotonin	Sigma Aldrich	H7752

Bile Acids

glycochenodeoxycholic	Sigma Aldrich	G0759
glycocholic	Calbiochem	360512
glycodeoxycholic	Calbiochem	361311
taurochenodeoxycholic	Sigma Aldrich	T6260
taurocholic	Calbiochem	580217
taurodeoxycholic	Calbiochem	580221

AMC: Herman Ten Brink, PhD. Amsterdam University Medical Center. Amsterdam, Netherlands. https://www.vumc.com/departments/clinical-chemistry/metabolic-laboratory/organic-synthesis-laboratory.htm
Cambridge Isotopes. Tewksbury, MA, USA. https://www.isotope.com
CDN Isotopes. Pointe-Claire, Quebec, Canada. https://cdnisotopes.com
Dr. Schircks Laboratories. Bauma, Switzerland. http://www.schircks.ch/company/schircks_info_frame1.htm
FUJIFILM Wako Chemicals USA Co.,Richmond, VA, USA. https://labchem-wako.fujifilm.com/us/category/00439.html
Millipore Sigma (Sigma Aldrich, Fluka, calbiotech). St. Louis, MO, USA. https://www.sigmaaldrich.com
MP Biomedicals (formerly ICN). Irvine, CA, USA. https://www.mpbio.com/
Santa Cruz Biotechnology. Dallas, TX, USA. https://www.scbt.com/home
Toronto Research Chemicals. Ontario, Canada. https://www.trc-canada.com/
UAM: Ernesto Brunet, PhD. Universidad Autónoma de Madrid. Madrid, Spain. http://qorganica.com/lumila/standards/standards.htm
