# Supplementary material for: Identification of pathognomonic purine synthesis biomarkers by metabolomic profiling of adolescents with obesity and type 2 diabetes
Source: PLoS One. 2020 Jun 26;15(6):e0234970. doi: 10.1371/journal.pone.0234970 (PMC7319336; doi:10.1371/journal.pone.0234970)
Supplement: S3 Table — Urine metabolite concentrations were measured as mmol/mmol creat and log2-transformed for subsequent analysis. Data here are reported as mean and 95% confidence intervals after inverse log transformation. T2D signature: Post-hoc Tukey T2D>OB and T2D>NW, or T2D<NW and T2DNW and OB>NW, or T2D<NW and OB<NW. (PDF) [file pone.0234970.s003.pdf]

	HMDB ID	Corrected *P*	Urine metabolites (95% CI) NW	OB	T2D	
**Signature for Type 2 diabetes**						
SAICA-riboside	HMDB00797	9.50E-08	1.18 (1.02, 1.34)	1.67 (1.42, 1.93)	2.15 (1.98, 2.32)	▲
Aconitic acid	HMDB00072	0.0003	3.42 (3.27, 3.58)	3.27 (3.03, 3.61)	4.37 (4.04, 4.70)	▲
Betaine	HMDB00043	0.0004	1.85 (1.47, 2.23)	1.89 (1.15, 2.63)	3.74 (3.17, 4.32)	▲
Dimethylglycine	HMDB00092	0.0004	1.60 (1.34, 1.86)	1.51 (1.07, 1.95)	2.58 (2.29, 2.86)	▲
Valine	HMDB00883	0.0011	1.75 (1.59, 1.92)	1.59 (1.11, 2.07)	2.69 (2.35, 3.03)	▲
Cystine	HMDB00192	0.0016	2.64 (2.46, 2.82)	2.01 (2.54, 3.28)	3.48 (3.19, 3.77)	▲
Thymidine	HMDB00273	0.0016	-6.50 (-6.57, -6.43)	-6.39 (-6.45, -6.32)	-6.19 (-6.30, -6.09)	▲
Leucine	HMDB00687	0.0016	1.48 (1.31, 1.65)	1.02 (0.33, 1.71)	2.31 (1.97, 2.65)	▲
Tyrosine	HMDB00158	0.0016	3.17 (2.98, 3.36)	3.04 (2.56, 3.53)	3.86 (3.60, 4.13)	▲
Ornithine	HMDB00214	0.0016	-0.21 (-0.39, -0.03)	-0.30 (-0.72, 0.13)	0.72 (0.35, 1.09)	▲
Cinnamoylglycine	HMDB11621	0.0017	-0.21 (-0.78, 0.37)	-1.84 (-2.56, -1.11)	-3.81 (-6.62, -2.46)	▼
Phenylalanine	HMDB00159	0.0018	5.52 (5.36, 5.67)	5.23 (4.79, 5.67)	6.05 (5.84, 6.26)	▲
AICA-riboside	HMDB62424	0.0019	2.83 (2.67, 3.00)	3.03 (2.78, 3.28)	3.45 (3.23, 3.66)	▲
Tryptophan	HMDB00929	0.0019	2.00 (1.84, 2.15)	1.81 (1.39, 2.22)	2.59 (2.34, 2.83)	▲
3-hydroxybutyric acid	HMDB00357	0.0029	-0.82 (-1.29, -0.35)	-0.75 (-1.28, -0.23)	0.91 (0.30, 1.52)	▲
3-hydroxyisobutyric acid	HMDB00023	0.0033	3.67 (3.40, 3.94)	3.72 (3.27, 4.17)	4.63 (4.27, 4.99)	▲
Mevalonic acid	HMDB00227	0.0041	0.51 (0.30, 0.72)	0.62 (0.27, 0.96)	1.37 (1.03, 1.71)	▲
2-Oxoisovaleric acid	HMDB00019	0.0054	-4.01 (-4.35, -3.68)	-3.92 (-4.27, -3.57)	-2.97 (-3.39, -2.55)	▲
3-methyl-2-oxovaleric	HMDB00491	0.0068	-1.12 (-1.46, -0.78)	-1.29 (-2.04, -0.55)	0.26 (-0.38, 0.90)	▲
N-acetyltyrosine	HMDB00866	0.0139	-3.71 (-4.17, -3.25)	-3.99 (-4.64, -3.34)	-2.80 (-3.21, -2.38)	▲
Alanine	HMDB00161	0.0247	6.61 (6.39, 6.83)	6.41 (6.05, 6.78)	7.11 (6.84, 7.38)	▲
4-hydroxyproline	HMDB00725	0.0302	4.14 (3.81, 4.48)	4.27 (3.91, 4.63)	4.89 (4.57, 5.22)	▲
**Signature for obesity with or without diabetes**						
Isobutyrylglycine	HMDB00730	4.00E-05	-1.00 (-1.27, -0.73)	-2.34 (-2.91, -1.77) ▼	-2.57 (-3.08, -2.06)	▼
Isovalerylglycine	HMDB00678	4.00E-05	0.41 (0.13, 0.70)	-1.11 (-1.66, -0.55) ▼	-0.73 (-1.07, -0.39)	▼
Uracil	HMDB00300	0.0003	6.1 (5.85, 6.35)	5.09 (4.57, 5.61) ▼	5.2 (4.96, 5.43)	▼
Heptanoylglycine	HMDB13010	0.0011	-4.47 (-4.84, -4.10)	-5.45 (-5.91, .4.99) ▼	-5.44 (-5.76, -5.13)	▼
Tiglylglycine	HMDB00959	0.0045	0.98 (0.77, 1.20)	0.14 (-0.27, 0.56) ▼	0.46 (0.21, 0.71)	▼
3-Methylcrotonylglycine	HMDB00459	0.0094	-1.80 (-2.06, -1.53)	-2.66 (-3.15, -2.16) ▼	-2.44 (-2.74, -2.15)	▼
2-hydroxyadipic	HMDB00321	0.0140	-3.67 (-4.25, -3.08)	-2.37 (-2.96, -1.77) ▲	-2.05 (-2.40, -1.70)	▲
Glycodeoxycholic	HMDB00631	0.0353	-8.72 (-9.30, -8.14)	-6.95 (-7.45, -6.46) ▲	-6.87 (-7.60, -6.14)	▲
