# Supplementary material for: Identification of pathognomonic purine synthesis biomarkers by metabolomic profiling of adolescents with obesity and type 2 diabetes
Source: PLoS One. 2020 Jun 26;15(6):e0234970. doi: 10.1371/journal.pone.0234970 (PMC7319336; doi:10.1371/journal.pone.0234970)
Supplement: S4 Table — Plasma metabolite concentrations were measured as mM and log2-transformed for subsequent analysis. Data here are reported are mean and 95% confidence intervals after inverse log transformation. T2D signature: Post-hoc Tukey T2D>OB and T2D>NW, or T2D<NW and T2DNW and OB>NW, or T2D<NW and OB<NW (PDF) [file pone.0234970.s004.pdf]

			Plasma metabolites, 95% CI		
	**HMDB ID**	**Corrected *P***	**NW**	**OB**	**T2D**
**Signature for Type 2 diabetes**					
L-DOPA	HMDB00181	0.023	1.78 (1.31, 2.25)	2.54 (1.84, 3.24)	0.65 (-0.81, 0.94) ▼
**Signature for obesity with or without diabetes**					
Isoleucine	HMDB00172	0.0006	6.12 (6.03, 6.21)	6.48 (6.34, 6.62) ▲	6.62 (6.47, 6.77) ▲
Glutamic acid	HMDB00148	0.0006	6.33 (6.19, 6.48)	7.06 (6.86, 7.26) ▲	6.99 (6.68, 5.31) ▲
Valine	HMDB00883	0.0008	6.65 (6.55, 6.76)	7.02 (6.89, 7.16) ▲	7.82 (6.95, 7.24) ▲
Leucine	HMDB00687	0.0011	6.65 (6.55, 6.76)	7.02 (6.89, 7.16) ▲	7.10 (6.95, 7.24) ▲
SAICA-riboside	HMDB00797	0.0208	1.00 (0.75, 1.26)	1.48 (1.20, 1.75) ▲	1.71 (1.48, 1.93) ▲
Isobutyrylglycine	HMDB00730	0.0296	-7.20 (-7.98, -6.43)	-9.46 (-9.98, -8.95) ▼	-9.07 (-9.69, -8.46) ▼
3-Hydroxyisobutyric acid	HMDB00023	0.0296	4.25 (4.05, 4.44)	4.84 (4.63, 5.07) ▲	4.68 (4.42, 4.94) ▲
Xanthine	HMDB00292	0.0378	-0.96 (-1.21, -0.69)	-0.25 (-0.69, 0.19) ▲	-0.15 (-0.57, 0.28) ▲
2-Hydroxybutyric acid	HMDB00008	0.0392	4.73 (4.15, 5.31)	5.86 (5.51, 6.20) ▲	6.05 (5.75, 6.36) ▲
3-Methyl-2-oxovaleric acid	HMDB00491	0.0392	4.12 (3.82, 4.42)	4.54 (4.34, 4.73) ▲	4.70 (4.52, 4.88) ▲
Pyridoxal	HMDB01545	0.0390	-1.37 (-1.52, -1.21)	-0.88 (-1.14, -0.63) ▲	-0.97 (-1.22, -0.73) ▲
