# Supplementary figures and images for: Identification of pathognomonic purine synthesis biomarkers by metabolomic profiling of adolescents with obesity and type 2 diabetes
Source: PLoS One. 2020 Jun 26;15(6):e0234970. doi: 10.1371/journal.pone.0234970 (PMC7319336; doi:10.1371/journal.pone.0234970)

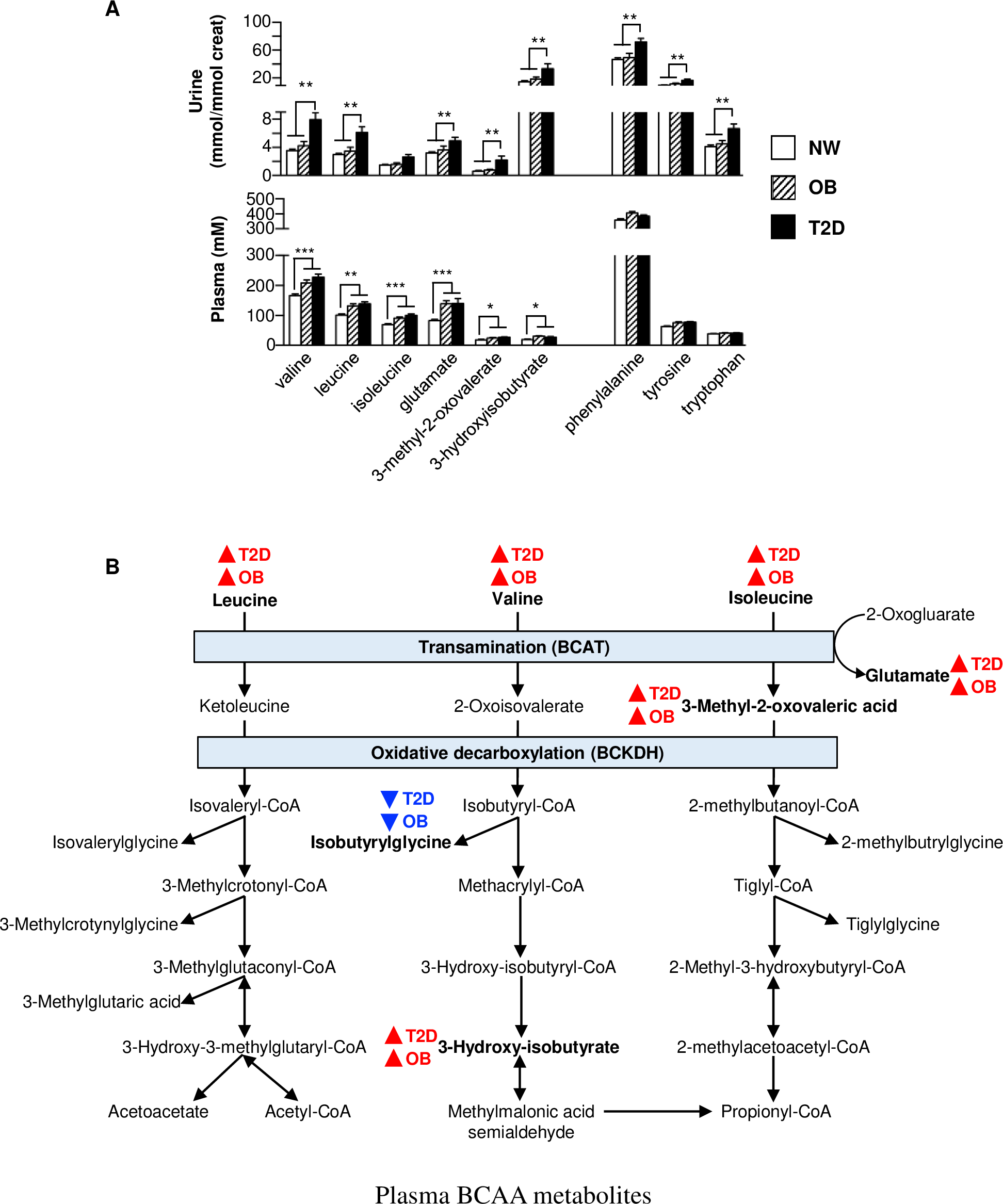

Supplement: S1 Fig — Comparison of urine and plasma BCAA metabolites associated with type 2 diabetes or obesity A) Urinary BCAAs and several of their degradation products are increased in T2D compared to OB and NW controls. In contrast, plasma BCAAs and their catabolites are increased in both T2D and OB groups compared to NW controls. B) BCAA degradation pathways are shown. Red triangle symbols with T2D indicate post-hoc Tukey T2D>NW, and red triangle symbols with OB indicate post-hoc Tukey OB>NW. Blue triangle symbols with T2D indicate post-hoc Tukey T2D<NW, and blue triangle symbols with OB indicate post-hoc Tukey OB<NW. (TIFF) [file pone.0234970.s005.tiff]

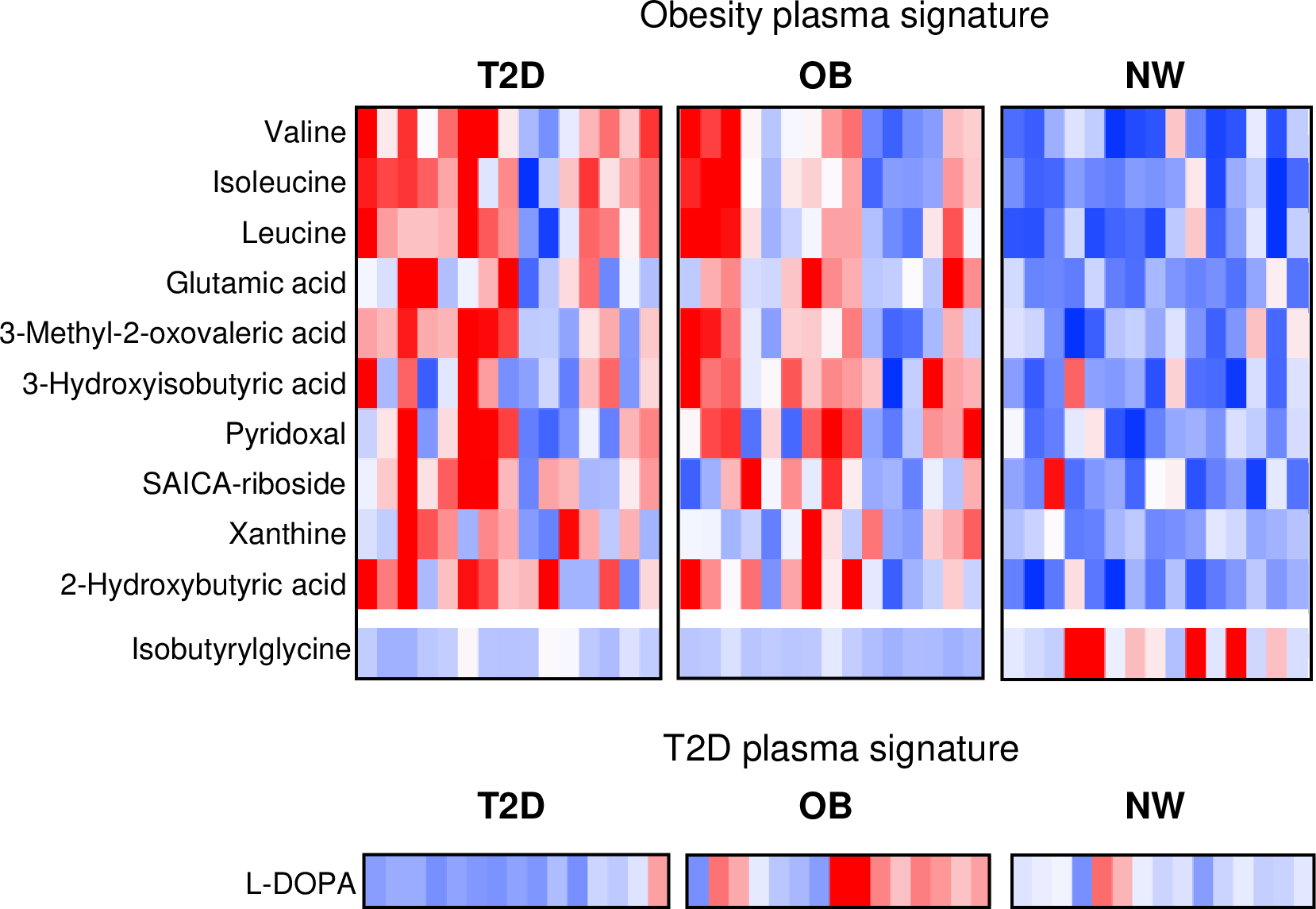

Supplement: S2 Fig — Signatures consists of metabolites that met a 5% FDR cutoff.Type 2 diabetes signature: Post-hoc Tukey T2D>OB and T2D>NW, or T2D<NW and T2DNW and OB>NW, or T2D<NW and OB<NW (TIFF) [file pone.0234970.s006.tiff]

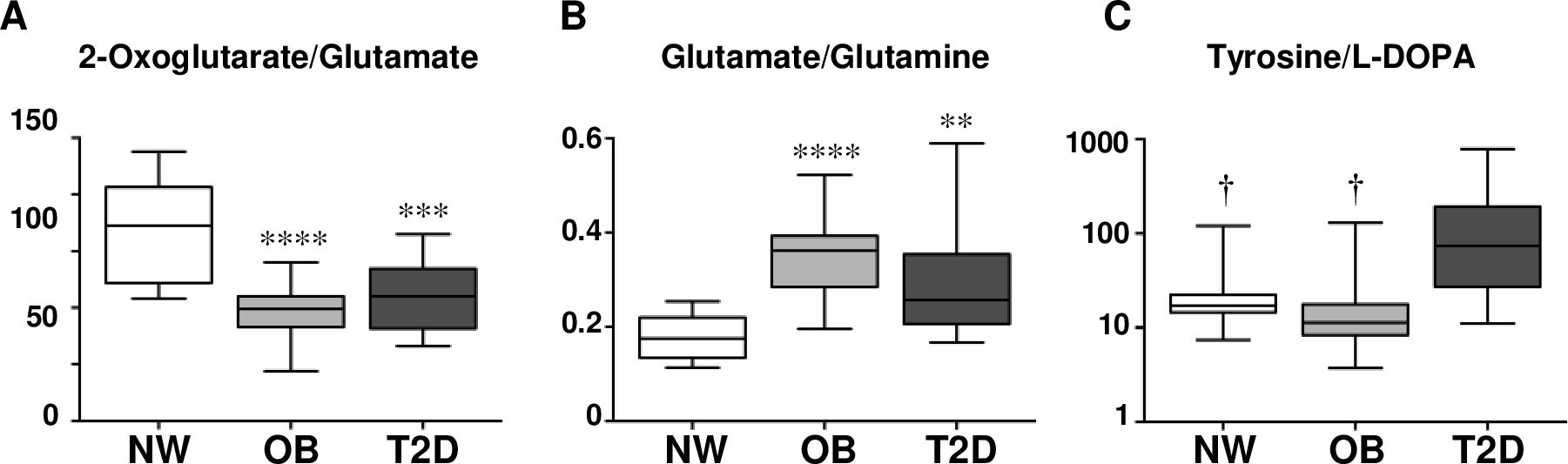

Supplement: S3 Fig — Metabolite ratios used to infer enzyme activity A) 2-Oxoglutarate to glutamate ratio: branched-chain amino acid transaminase activity. B) Glutamate to glutamine ratio: glutamine synthetase activity. C) Tyrosine to L-DOPA ratio: tyrosine hydroxylase activity. **p<0.01 vs. NW, ***p<0.001 vs. NW, ****p<0.0001 vs NW, †p<0.05 vs. T2D. (TIFF) [file pone.0234970.s007.tiff]

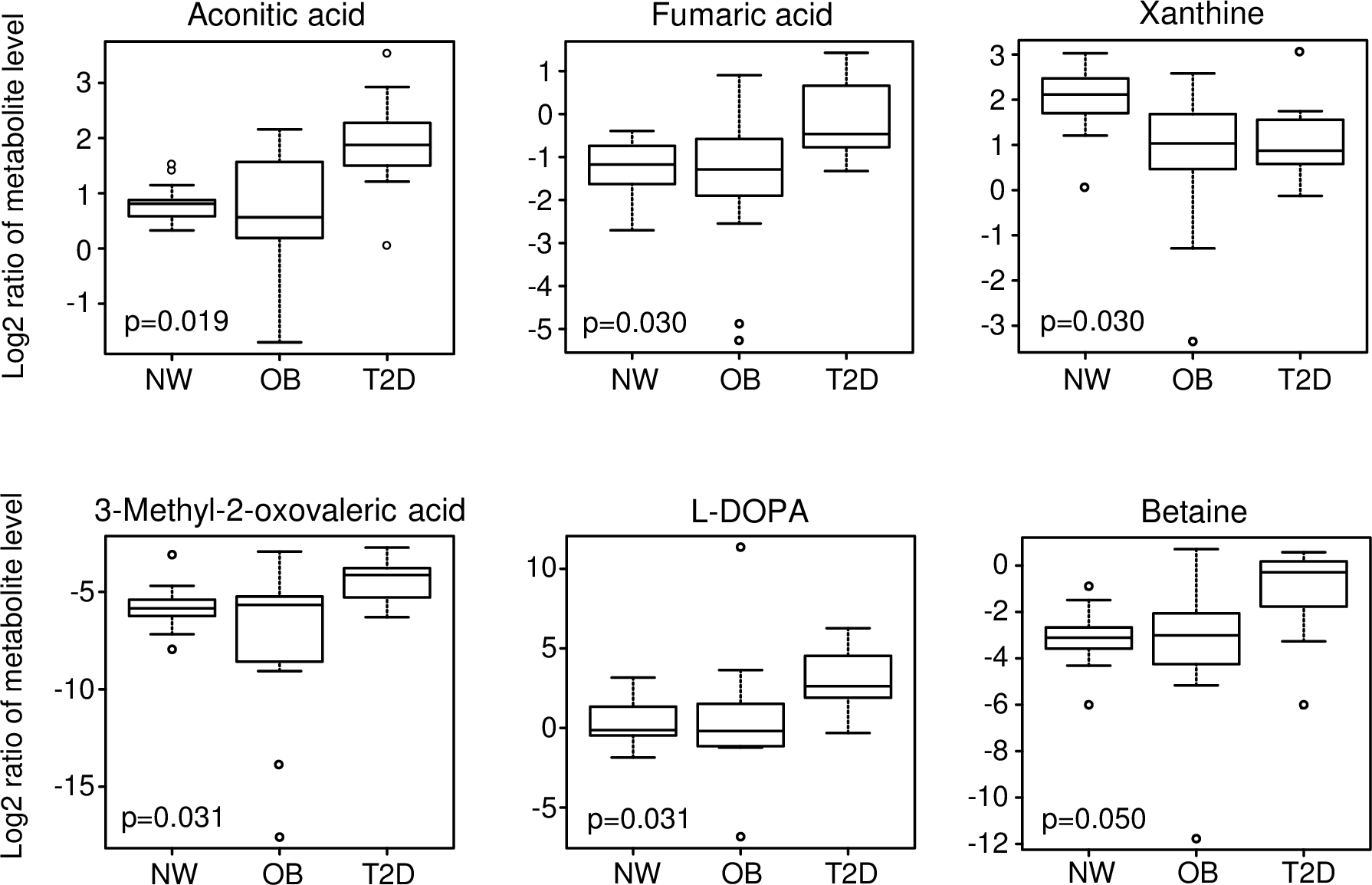

Supplement: S4 Fig — Post-hoc Tukey T2D>NW and T2D>OB at p≤0.05. (TIFF) [file pone.0234970.s008.tiff]

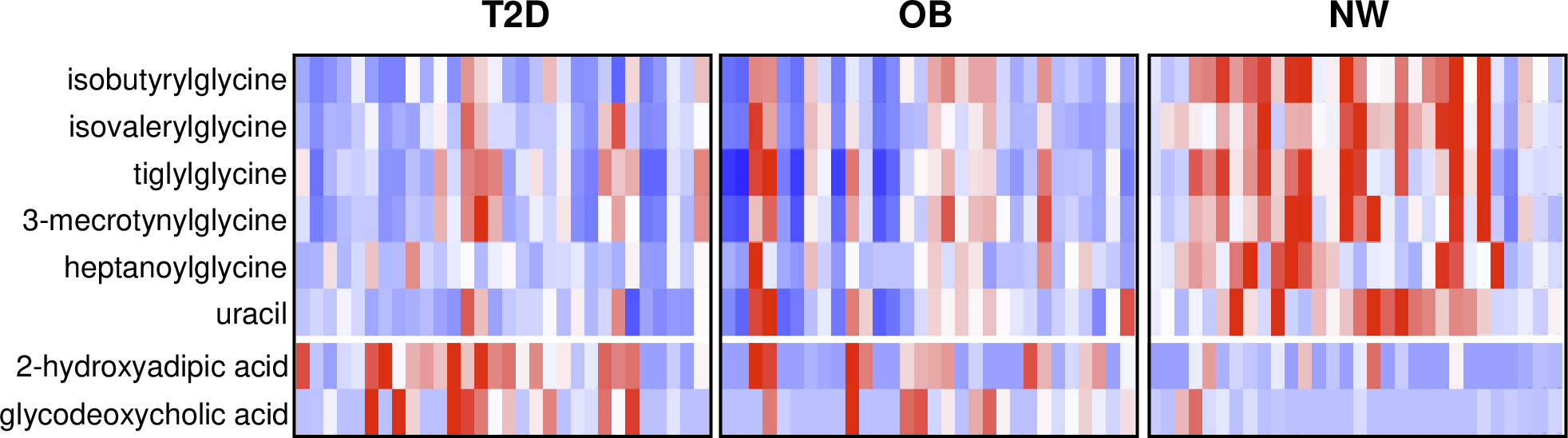

Supplement: S5 Fig — Obesity signature: Post-hoc Tukey T2D>NW and OB>NW, or T2D<NW and T2D<OB. (TIFF) [file pone.0234970.s009.tiff]

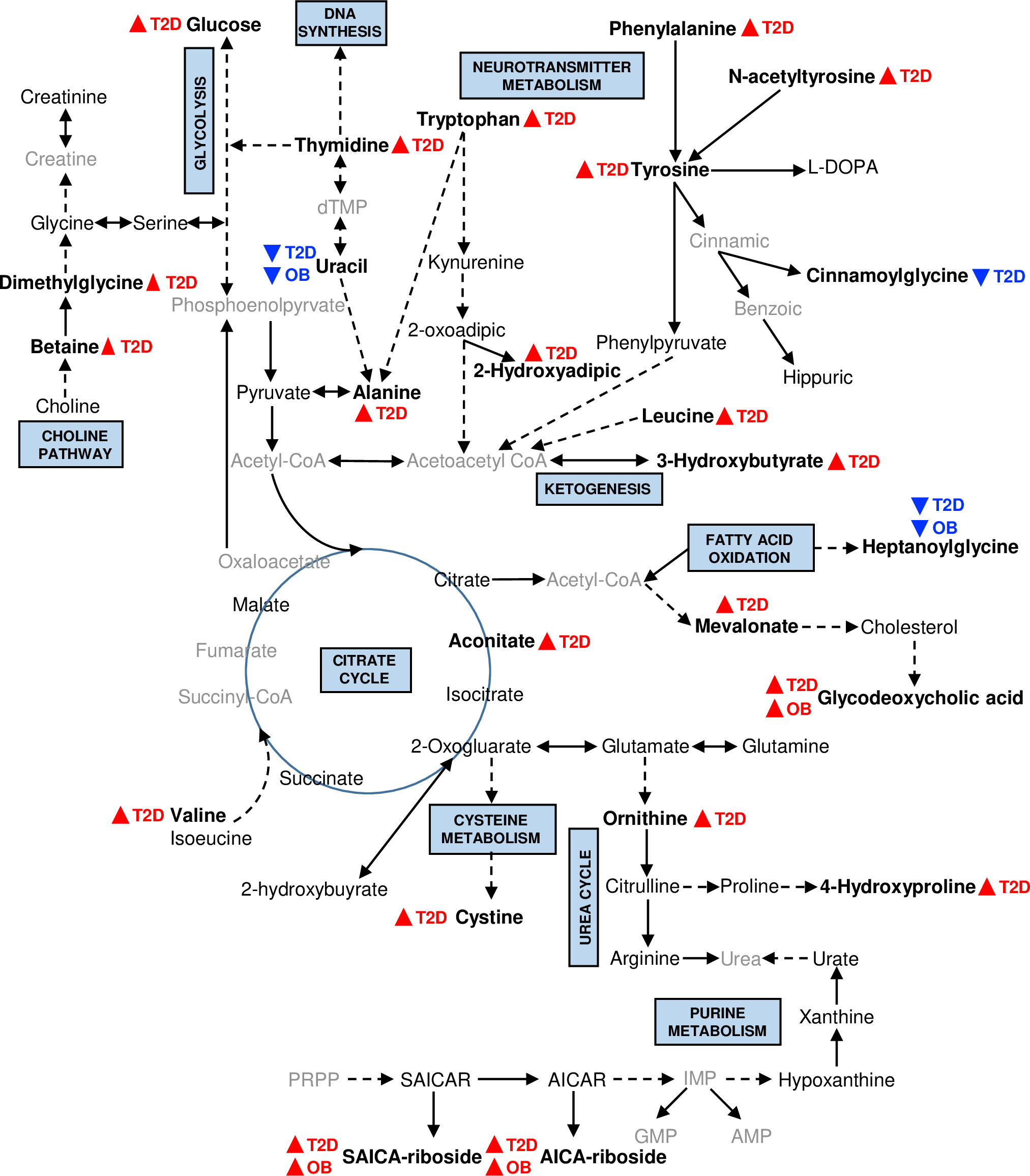

Supplement: S6 Fig — Red triangle symbols with T2D indicate post-hoc Tukey T2D>NW, and red triangle symbols with OB indicate post-hoc Tukey OB>NW. Blue triangle symbols with T2D indicate post-hoc Tukey T2D<NW, and blue triangle symbols with OB indicate post-hoc Tukey OB<NW. (TIFF) [file pone.0234970.s010.tiff]
